# The implicit beliefs and implicit behavioral tendencies towards smoking-related cues among Chinese male smokers and non-smokers

**DOI:** 10.1186/s12889-019-7319-7

**Published:** 2019-07-25

**Authors:** Lei Ren, Long-Biao Cui, Chen Chen, Xiaojun Dong, Zhongying Wu, Yidi Wang, Qun Yang

**Affiliations:** Department of Clinical Psychology, Fourth Military Medical University, No. 169 West Changle Road, Xi’an, 710032 China

**Keywords:** Implicit association test, Implicit beliefs, Implicit behavioral tendencies, Smoking behavior

## Abstract

**Background:**

The dual-process theory is central to several models of addiction, implying the importance of automatic processes in the maintenance and development of addiction. Implicit beliefs are traces of previous experience which relate to the representation in cognition. Implicit behavioral tendencies are traces of previous experience which relate to the representation in behavioral tendencies. In this study, we aim to provide behavioral evidence for implicit beliefs and implicit behavioral tendencies towards smoking-related cues among Chinese male smokers and non-smokers. We also examine the relationships among implicit beliefs, implicit behavioral tendencies and smoking behaviors of smokers.

**Methods:**

In order to achieve these goals, we used an implicit association test (IAT) to measure implicit beliefs and implicit behavioral tendencies simultaneously. Thirty-nine smokers and twenty-five non-smokers were tested, using smoking-related words and images, as well as neutral words and images as stimuli.

**Results:**

Our analysis shows significant differences in smokers’ and non-smokers’ implicit beliefs and behavioral tendencies (t_62_ = 3.494, *p* < 0.001; t_62_ = 5.034, *p* < 0.001). In the group of smokers, implicit beliefs and implicit behavioral tendencies were positively correlated with each other (r = 0.460, *p* < 0.01). In addition, smokers’ scores for implicit behavioral tendencies are negatively correlated with the number of cigarettes smoked per day (r = − 0.51, *p* < 0.001).

**Conclusions:**

This study suggests that implicit beliefs and behavioral tendencies toward smoking-related cues vary significantly between Chinese male smokers and non-smokers. In addition, there is a positive correlation between implicit beliefs and behavioral tendencies within smokers. It also shows for the first time that the implicit behavioral tendencies are related to smoking behaviors. Our results may be considered as references for smoking cessation interventions focused on changes at the implicit level, and they provide a new perspective for measuring different dimensions of implicit attitudes by an IAT. This finding might promote the development of the network theory of implicit attitudes.

**Electronic supplementary material:**

The online version of this article (10.1186/s12889-019-7319-7) contains supplementary material, which is available to authorized users.

## Background

The tobacco epidemic is one of the biggest public health threats worldwide. In fact, tobacco is regarded as one of the most addictive substances, with 32% of users dependent on it, among the addictive drugs, and more than 7 million people die from tobacco-related diseases every year [1]. In particular, there are more than 300 million smokers in China, which has the largest population of smokers in the world. Also, more than 1 million people die from smoking-related diseases every year, and about 100 thousand people die from secondhand smoke in China. Despite the awareness of its negative consequences on health and intensive prevention and control efforts, this problem still seems to be persistent [2]. The Chinese government has taken many actions such as informing consumers through the use of warning labels and mass media campaigns, raising prices of tobacco through taxation, and developing new tobacco control policies. However, the smoking rate in China still remains high [3]. Governments and civil society around the world recognize the risks posed by cigarette addiction, and China is not alone in seeking to mitigate tobacco’s harmful effects on its society. Our research may provide useful insight for those seeking to develop policy that promotes smoking cessation.

Dual-system models of addiction are concerned with both automatic and controlled systems [4]. These two systems are supposed to have distinctive contributions to smoking behaviors [5]. The automatic system is fast, effortless, irrational, uncontrolled. As such, it is critical we avoid overlooking its role in the maintenance and development of addiction [6, 7]. Smoking as an addictive behavior is significantly affected by automatic processes. Therefore, improving our understanding of the automatic processes that lead to smoking behaviors could be a crucial step in developing a understanding of how to overcome the addiction.

The automatic system suggests that when smokers encounter smoking-related stimuli, their implicit evaluative processes and implicit representations interact with each other to generate implicit attitudes, which then lead to implicit behaviors [8–10]. Recent research suggests that an automatic system may include not only associations but complex smoking-related beliefs as well [11].

Smokers have various cognitions as they recall their past experiences that mediate favorable or unfavorable attitudes toward smoking. Smokers expect to handle stress by smoking, and they believe that smoking is associated with social outcomes such as feeling relaxed, attractive and uncontrolled [12–14]. Because of the cultural background, Chinese smokers believe that smoking represents maturity and fascination, and a man who does not smoke is considered unmanly [15]. Also, sharing cigarettes is a social behavior intended to show hospitality [16]. These factors weaken correct cognitions on tobaccos and play important roles in maintaining and developing smoking behaviors [17]. Smokers are also aware of the fact that smoking has health-damaging consequences [13, 14]. However, it seems that smokers ignore the negative consequences of smoking and justify their behaviors with positive outcomes [17]. When these thoughts and opinions are deeply rooted, the implicit representations are formed. In this case, the relatively positive implicit cognition is automatically activated as soon as they encounter smoking-related cues. Such implicit cognition is often (if not always) hard to notice, making the behaviors it motivates more difficult to control. Addictive behaviors such as smoking can be effectively cured by cognitive behavioral therapy and acceptance and commitment therapy, which also indicates that irrational cognition exists among smokers [18–20]. According to Greenwald’s definitions of implicit attitude and dual-attitude model [8–10], the implicit beliefs towards smoking are inaccurately identified traces of previous experience which focus on the representation in cognition about smoking; and the implicit behavioral tendencies towards smoking are inaccurately identified as traces of previous experience which focus on the representation in behavioral tendencies about smoking. Therefore, we inferred that smokers have positive implicit beliefs (cognitively based attitude) toward smoking and that they would show an automatic approach association in implicit behavioral tendencies because of the constant behavioral reinforcement and implicit beliefs. Additionally, research on alcohol consumption demonstrates that alcohol-dependent patients have stronger alcohol-approach association scores on the Implicit Association Test (IAT, a computer-based, response-mapping task that is designed to measure the implicit association between target concepts) when compared with control group, and this difference in scores is associated with drinking behaviors [21]. Thus, we hypothesize the implicit behavioral tendencies scores in the present study are similarly associated with smoking behavior.

The most extensively utilized implicit measure for this purpose is the IAT [22, 23]. The IAT measures the strength of associations between targets and attributes. In this paradigm, it is assumed that closely related concepts share the same reaction key. This, in turn, makes association between closely related concepts a simpler task for the mind, requiring less mental effort and resulting in a quicker response. Although there is some debate about the potential cognitive processes that produce results on IAT, a considerable number of investigators support the constructive validity of IAT as a measure of cognitive associations which are automatically activated in many cases, including the use in addiction research [24–26]. The IAT measures associations in long-term memory between words and specific concepts. It may reveal processes related to tobacco dependence that are both unique to the addiction, and held in common with other addictions [21]. In the present study, we put implicit beliefs and implicit behavioral tendencies in one IAT to exclude confounding effects, measure the relationships between implicit beliefs and implicit behavioral tendencies, and quantify their respective implicit effects. Meanwhile, we applied a modified analysis method based on Greenwald, et al’s scoring algorithm, and, adapted it to this study [27].

Therefore, the first aim of this study was to explore implicit beliefs and behavioral tendencies towards smoking cues between smokers and non-smokers by using the IAT. The second aim was to explore the relationships among implicit beliefs, implicit behavioral tendencies and smoking behaviors.

## Methods

### Ethics statement

The present study is a cross-sectional study. The independent Ethics Committee, First Affiliated Hospital of Air Force Military Medical University granted ethical approval for the present study. All participants signed the written informed consent forms prior to participation. They were assured that there is no risk of harm from this experiment, and were informed that the experiment contained a computer-based task and one scale. The results would be strictly confidential. In addition, participants were informed that they could opt out of the experiment at any time. All participants were compensated for their time (about 15 US dollars) and thanked for their participation.

### Participants

Participants include 39 smokers and 25 non-smokers recruited from the Air Force Military Medical University, who were male undergraduate students majoring in clinical medicine. To control the gender effect, we only chose men as participants. As most smokers in China are male, this sample aims to focus the research on the population where results will be most meaningful. After a brief interview screening, people with past or current drug consumption habits (other than moderate alcohol and cigarette) or major medical issues were excluded. For the purposes of our study, non-smokers are considered to be those who have either never smoked in the past, or who have consumed fewer than 10 cigarettes throughout their lifetime. Participants were asked to abstain from smoking for 2 h prior to taking the test, to avoid floor or ceiling effects related to nicotine cravings (or the absence thereof) [28]. Both groups were at similar age and level of education (see Table 2 for demographic data).

### Demographics and smoking characteristics

The primary investigation was composed of an assessment of demographics and of smoking characteristics, such as frequency of smoking and years of smoking. Respondents’ degree of nicotine dependence was assessed with the Fagerström test for nicotine dependence (Additional file 1) [29]. It is a 6-item self-report that is widely used, reliable, and well-validated for capturing the degree of nicotine dependence [30, 31].

### Measurement of implicit attitudes

A computer-based response-latency method was implemented using the Implicit Association Test [22], to assess automatic features of implicit attitudes toward smoking. In accordance with previous research, this test used similar stimuli and procedures in order to maximize consistency with, and comparability to, previous findings (see Table 1) [5, 25, 26]. The target categories were labeled as “smoking” (including six pictures related to smoking (e.g. an image of cigarettes with a lighter, an image containing a lit cigarette) and “shapes” (including six pictures of line-drawn, geometric shapes with no direct link to smoking behaviors). The attribute categories could be labeled as “positive words” (including three words representing implicit beliefs and three words representing implicit behavioral tendencies) or as “negative words” (including three words representing implicit beliefs and three words representing implicit behavioral tendencies). The image size was set to 20% of the width and height of a 14-in. computer screen. The background in images were consistent, and the brightness levels were constant between the ‘smoking’ and ‘shape’ pictures. Letters were sized 5% of the screen’s dimensions, and green text was used to present attribute words [5].

**Table 1:** Stimulus material

Target categories	Smoking	Six pictures related to smoking
	Shapes	Six pictures of line-drawn shapes
Attribute categories	Positive words	Three positive cognition words
		Three positive behavioral tendency words
	Negative words	Three negative cognition words
		Three negative behavioral tendency words

Participants were tasked with responding to the stimuli that appeared in the center of the screen, as quickly as possible, according to the labels presented in the upper right or left corner of the screen, by pressing the letter E (left) or I (right) on the keyboard. Label letters were set to 3.5% of the dimensions of the display. The ‘smoking’ and ‘shapes’ categories were labelled with white text, while the “positive” and “negative” word categories were labelled with green text [5]. The IAT was programmed in E-prime 2.0 and consisted of seven phases: (1) 12 practice trials were used to categorize word stimuli into “positive words” or “negative words” and each word appeared randomly once; (2) 12 practice trials were used to categorize imagery into “smoking” or “shapes,” and each picture appeared randomly once; (3) and (4) 48 practice, 96 experimental trials were used to categorize word and picture stimulus pairs into one of the combined categories (such as smoking + positive words vs. shapes + negative words; each picture and attribute word appeared randomly twice in the practice phase and four times in the experimental phase); (5) the 12 practice trials were repeated in phase 2, with the locations of category labels (shapes vs. smoking) switched between left and right on the display; and (6) and (7) the 48 practice and 96 experimental trials in phase 3 and 4 were repeated, with the locations of the combined categories switched on the display, as well (eg, shapes + positive words vs. smoking + negative words). For each trial, the stimulus remained on the display until the participant made their selection with the keyboard. The next stimulus would appear if a participant made a correct button response while an “X” would appear as a feedback if a participant made an incorrect button response. The presentation order of paired categories was counterbalanced. The duration of all tasks for each participant was about 12 min. These tasks presumed that, when target and attribute category pairs agreed with participants’ automatic associations, stimuli would be easier to classify, and thus, classification should occur more rapidly. Likewise, when target and attribute category pairs do not coincide with participants’ automatic associations, response time should increase. In this study, we used one IAT to measure implicit beliefs and behavioral tendencies and applied a modified analysis method. This method is generally consistent with the scoring algorithm recommended by Greenwald and colleagues, with one change in calculating specific trials rather than all trials [27]. The D scoring algorithm recommended by Greenwald uses all trials at phases 3 and 4, 6 and 7 of IAT as raw data and perform specific calculations and transformations on the data. The resulting IAT effect, D score, has a similar interpretation as an effect-size measure. For our study, we picked some related trials from all trials and did the same calculations and transformations as the D scoring algorithm recommended by Greenwald to control deviations of results caused by common trials. For example, when calculating D score for implicit beliefs, we only used trials that represented implicit beliefs and excluded other trials such as words that represented implicit behavioral tendencies and target pictures to avoid their effects as joint terms. A higher positive D-score represents more negative automatic associations toward smoking. In addition, participants with more than 30% error rates in the main implicit association test were excluded in our data analyses. This includes trials in which latencies were < 300 ms or trials in which latencies were > 10000 ms, as these responses are considered either “too fast” or “too slow” (respectively). Internal consistency (Chronbach’s α) was measured at 0.88, and was calculated using response time for each experimental trial [5].

Our pilot study utilized four steps to compile a list of valid attribute words reflecting the implicit beliefs and behavioral tendencies toward smoking. First, 195 informants were tasked with writing down as many words as possible to reflect their responses toward smoking on papers, and 153 words consisting of two Chinese characters each (e.g., wei-hai, ju-jue) were obtained in this step. Then, another 39 undergraduates who majored in psychology classified these words into two categories: words related to cognitive evaluations and words related to behavioral reactions. Words that were classified into the same category more than half of the time by informants moved on to the next step: 42 words related to cognition and 34 words related to behavior were finally selected. Third, three separate groups of informants rated each word along the following categories: valence (*N* = 60; on a 7-point scale: 1 = definitely negative, 7 = definitely positive), arousal (N = 60; on a 7-point scale: 1 = definitely non-arousal, 7 = definitely arousal), and familiarity (N = 60; on a 7-point scale: 1 = definitely unfamiliar, 7 = definitely familiar). Lastly, we invited two Professors of Psychology, one Associate Professor of Linguistics, and twelve graduate students in Psychology department to select words according to the third step. After balancing arousal and familiarity, six positive words (three positive cognition words: mature, free, admirable; three positive behavioral tendency words: decompress, enjoy, proximity) and six negative words (three negative cognition words: harm, selfishness, pollution; three negative behavioral tendency words: reject, depart, contradict) were screened out in the last. The valence of positive and negative words varied significantly (words related to cognition: t = 23.85, *p* < 0.001; words related to behavior: t = 26.13, *p* < 0.001). In addition, informants who participated in the pilot study were excluded from the main experiment [32].

### Statistical analyses

The data were analyzed using SPSS 25 (IBM, Armonk, NY, USA). Significance levels were set at an alpha of 0.05 (two-tailed), and effects with a significance level of *p* < 0.05 were considered as trends.

### Group comparisons

All D scores were distributed normally in each group (Kolmogorov-Smirnov test: all *p* > 0.094). Variances of two groups were equal, according to Levene’s test (*p* = 0.742 for implicit beliefs; *p* = 0.125 for implicit behavioral tendencies).

Implicit beliefs, implicit behavioral tendencies, age (years) and level of education (years) were compared between two groups based on two-sample t-tests in our study.

### Correlations

For smokers, we performed Pearson’s r correlations among the implicit beliefs, implicit behavioral tendencies, cigarettes/day, years of smoking and FTND. The age (years) and the level of education (years) were not included in the correlation analysis because participants are basically the same in these two variables.

For non-smokers, we performed Pearson’s r correlations between implicit beliefs and behavioral tendencies.

## Result

### Smokers versus non-smokers

**Fig. 1:**
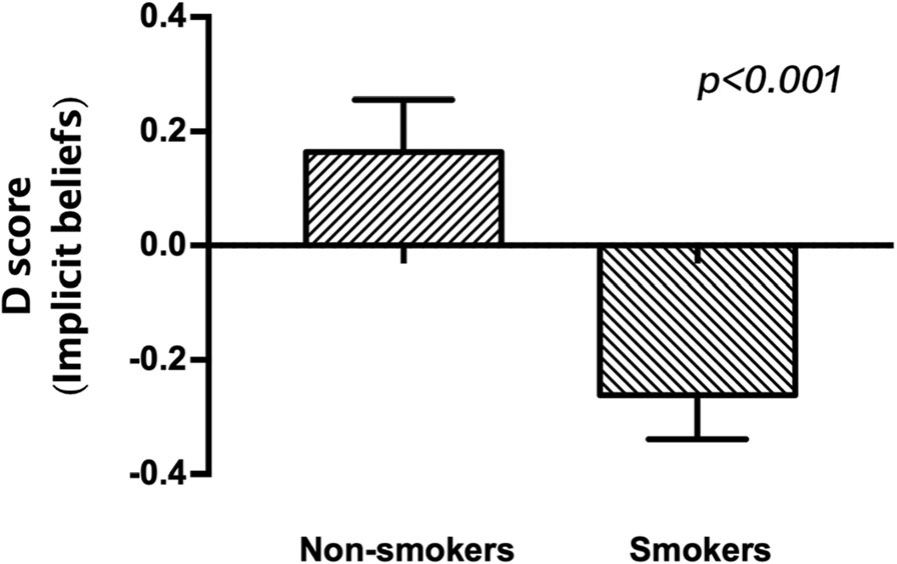
D-scores of implicit beliefs measured by the modified IAT, which were significantly different in smokers compared with non-smokers (*p* < 0.001)

**Fig. 2:**
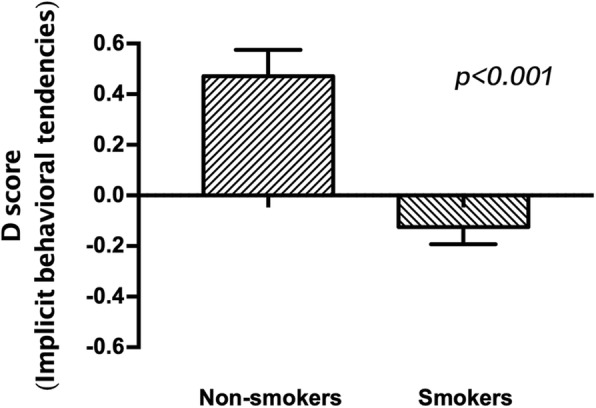
D-scores of implicit behavioral tendencies measured by the modified IAT, which were significantly different in smokers compared with non-smokers (*p* < 0.001)

As expected, implicit beliefs measured by the modified IAT were significantly different within smokers compared to non-smokers, and the D score of implicit beliefs was higher for non-smokers compared to smokers (t_62_ = 3.494, *p* < 0.001; Table 2; Fig. 1). The implicit behavioral tendencies were also significantly different between smokers and non-smokers, and the D score of implicit behavioral tendencies was higher for non-smokers compared to smokers (t_62_ = 5.034, *p* < 0.001; Table 2; Fig. 2). Moreover, there was no significant difference in age and level of education between smokers and nonsmokers.

**Table 2:** Group characteristics (Mean ± SD) and behavioral data

	Non-smokers	Smokers
Characteristic	*n* = 25	*n* = 39
Age(years)	20.12 ± 0.97	20.77 ± 1.25
Level of education(years)	14.36 ± 0.48	14.62 ± 0.49
Years of smoking	/	4.94 ± 1.92
Cigarettes/day	/	9.87 ± 6.27
FTND	/	2.38 ± 1.85
Implicit beliefs(D score)	0.16 ± 0.46	−0.26 ± 0.48 ***
Implicit behavioral tendencies(D score)	0.47 ± 0.52	− 0.13 ± 0.42 ***

***Significantly different from non-smokers at *p* < 0.001

Table 2 summarizes demographics, smoking characteristics and behavioral differences between the smokers and non-smokers.

### Correlations

**Fig. 3:**
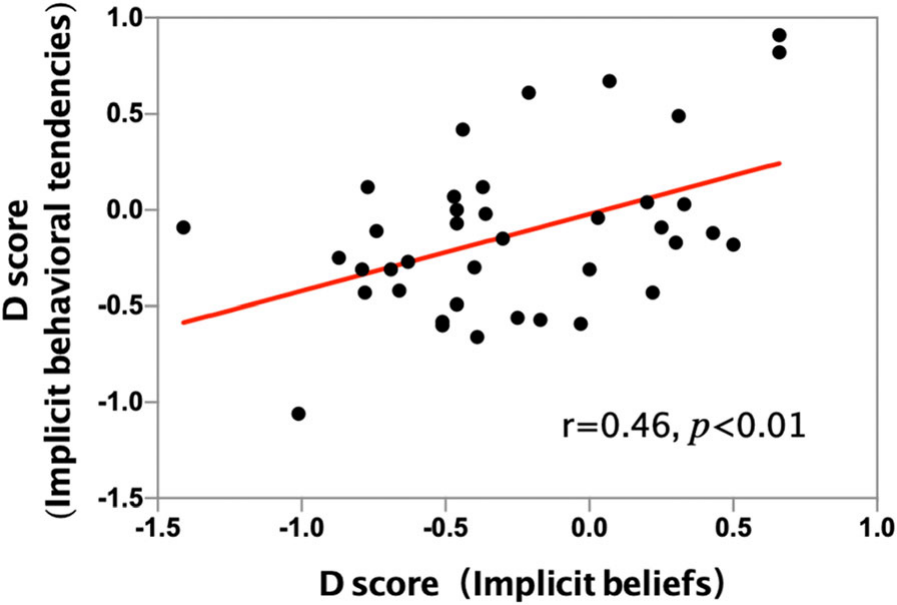
Implicit beliefs and implicit behavioral tendencies were positively correlated in the smoking group (*r* = 0.46, *p* < 0.01)

In the group of smokers, as the D-score of implicit beliefs increased, the D-score of implicit behavioral tendencies also increased (r = 0.460, *p* < 0.01; Table 3; Fig. 3). But this was not the case for the non-smokers group (r = − 0.13, *p* = 0.54).

**Table 3:** Correlation matrix of relevant variables in smokers

	Years of smoking	Cigarettes/day	FTND	IB-D score	IBT-D score
Years of smoking	1	0.33*	0.33*	0.02	−0.19
Cigarettes/day		1	0.75***	−0.14	−0.51***
FTND			1	−0.13	−0.38*
IB-D score				1	0.46**
IBT-D score					1

* *p* < 0.05, ** *p* < 0.01, *** *p* < 0.001. Abbreviations: IB Implicit beliefs, IBT Implicit behavioral tendencies

**Fig. 4:**
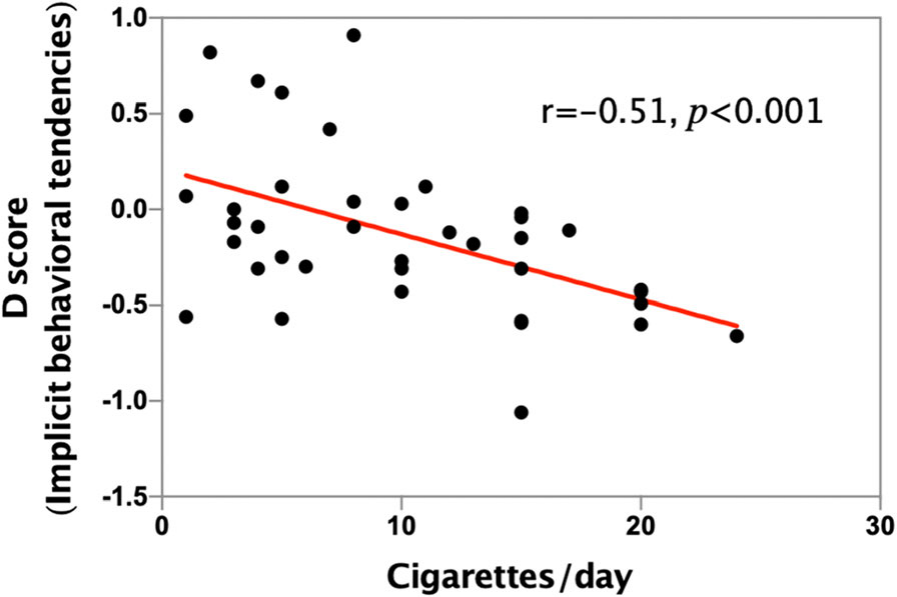
Cigarettes/day and implicit behavioral tendencies were negatively correlated in the smoking group (*r* = − 0.51, *p* < 0.001)

**Fig. 5:**
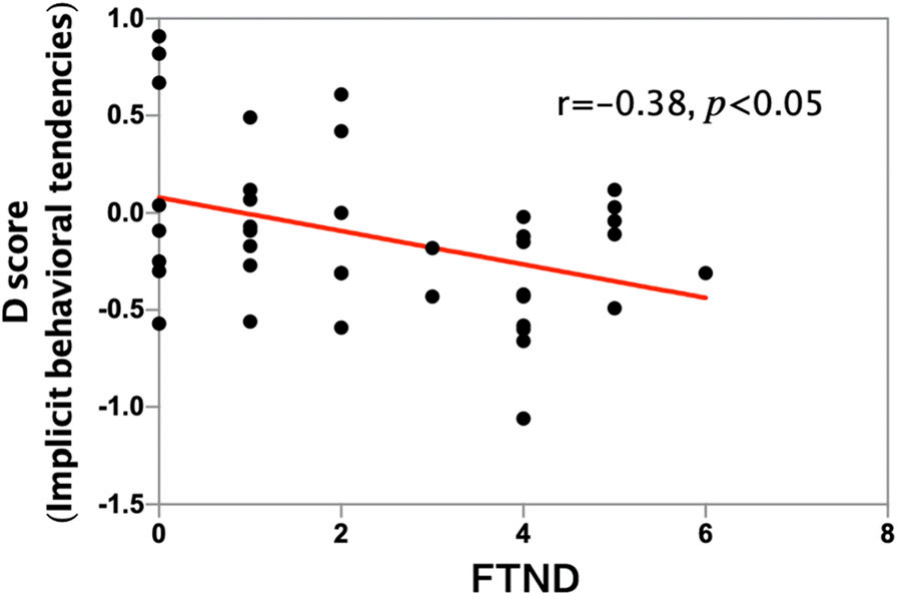
The FTND and implicit behavioral tendencies were negatively correlated in the smoking group (*r* = − 0.38, *p* < 0.05)

There was a negative correlation (which was statistically significant) between the implicit behavioral tendencies and cigarettes/day (*r* = − 0.51, *p* < 0.001; Table 3; Fig. 4). There was also a negative correlation between implicit beliefs and cigarettes/day (*r* = − 0.14, *p* = 0.41), though it was not statistically significant. The FTND and cigarettes/day were positively correlated with each other (*r* = 0.75, *p* < 0.001). Furthermore, implicit behavioral tendencies correlated with FTND (*r* = − 0.38, *p* < 0.05; Table 3; Fig. 5), but it did not have a significant difference between implicit beliefs and FTND (*r* = − 0.13, *p* = 0.42). There was a positive correlation between years of smoking and cigarettes/day (*r* = 0.33, *p* < 0.05). There was also a positive correlation between years of smoking and FTND (*r* = 0.33, *p* < 0.05). Years of smoking were correlated with implicit behavioral tendencies, but there was no significant difference (*r* = − 0.19, *p* = 0.24). This was the case with years of smoking and implicit beliefs as well (r = 0.02, *p* = 0.89).

A correlation matrix showing correlation coefficients among these variables is provided in Table 3.

## Discussion

This study for the first time puts implicit beliefs and behavioral tendencies together in one IAT. The results demonstrate that both implicit beliefs and implicit behavioral tendencies significantly differed between smokers and non-smokers. Specifically, smokers have closer associations between smoking and positive beliefs-related words, while non-smokers have closer associations between smoking and negative beliefs-related words. Similarly, smokers have closer associations between smoking and positive behavioral tendencies-related words while non-smokers have closer associations between smoking and negative behavioral tendencies-related words.

It has been reported that smokers have explicit irrational cognitions, especially young smokers in China. Smoking behavior is perceived as stylish, mature, and brings benefits to social interactions [15]. Therefore, they selectively ignore and deny the harmful effects of smoking on the health of themselves and the people around them [33]. These cognitions reduce the awareness of tobacco hazards and form the smoking behavior [12–14]. However, few studies have studied irrational cognitions on an implicit level. Our present study finds that smokers have positive implicit beliefs towards smoking versus non-smokers’ negative implicit beliefs towards smoking. Such results show that smokers have irrational cognitions on an implicit level, because of the constant reinforcement of explicit irrational cognitions.

As for the implicit behavioral tendencies, our present study is similar to the approach-avoidance IAT (‘approach’ and ‘avoidance’ labels are used to assess automatic approach/avoidance tendencies) which is not widely used and mostly focuses on alcohol-dependence [7, 34–36]. Previous research shows a consistent relationship between automatic approach association (as measured by the approach-avoidance IAT) and drug usage [11]. It is worth mentioning that the result of alcohol-approach association is negative rather than positive, which means negative implicit behavioral tendencies for alcohol-dependent patients [21]. However, implicit behavioral tendencies for smokers are positive in our study. Reasons for this phenomenon to happen can be that smoking is more likely to repeat in daily life so that constant reinforcement will show up more often, or that attribute words we chose may be more sensitive to the traces of memory of smokers about smoking. Another possible explanation might be that the way we calculate D value is slightly different. In addition, implicit beliefs are positively associated with implicit behavioral tendencies (r = 0.46, *p* < 0.01; Table 3; Fig. 3). Statistically, they might interact with each other, or be collectively influenced by a third factor. What we can predict is that when one changes, the other one will change similarly.

Moreover, implicit behavioral tendencies are negatively associated with frequency of smoking (r = − 0.51, *p* < 0.001; Table 3; Fig. 4), which means that the more smokers smoke, the stronger implicit smoking-approach tendencies they have. We speculate that persistent smoking behaviors would lead to implicit smoking-approach tendencies, which in turn will maintain smoking behaviors. Longitudinal studies are necessary to further research such interpretations. Thus, a better understanding of implicit behavioral tendencies in smoking behaviors could provide valuable information about mechanisms that maintain smoking behaviors and may guide the development of treatments to help smokers quit smoking. To some extent, it proves the effectiveness of cognitive behavioral therapy and approach-avoidance training in curing addictive behaviors [18, 25, 37, 38]. Our data also show that implicit behavioral tendencies are also associated with FTND (r = − 0.38, *p* < 0.05). FTND is an effective indicator of nicotine dependence [28–30], which implies that implicit tendencies are associated with the degree of tobacco dependence. We also notice that there is a negative correlation between implicit beliefs and smoking behaviors that is not statistically significant.

Finally, it is worth mentioning that the network method, which is becoming increasingly popular in psychological research (e.g. personality, emotion, intelligence and mental disorder), would fit well with the theory of implicit attitude [10, 39–42]. The implicit attitude is a vast network of representations, each concept (It can be considered that each word in this study represents a concept) is a node in the network and the edges between nodes represent the connection between different concepts. Everyone has their own unique network of implicit attitude because everyone has different characteristics on nodes and edges. So, the network method can fully explain the inconsistent results in measuring automatic processes among smokers [11, 43–46]. In addition, these nodes can further form several units. One of the feasible ways to develop a network theory of implicit attitude is to test as many units as possible in one IAT. In this study, we have two units, implicit beliefs and behavioral tendencies, together in one IAT and use a modified analysis method to measure implicit effects. However, the validity of our method needs to be further verified in subsequent experiments. A future study could look at more units, even down to each node. The present study might be able to promote the development of the network theory of implicit attitude and improve our understanding of the complexity of implicit attitude.

### Strengths and limitations

The present study explores implicit beliefs and behavioral tendencies towards smoking-related cues among smokers and non-smokers. This research deals with a topical public health issue and our findings might provide some guidelines for smoking cessation interventions from an implicit level. Words used in our IAT are derived from the designed survey, and may more accurately relate to respondents’ implicit beliefs and behavioral tendencies than those used in previous studies. In addition, the approach taken in our study is likely to contribute to the development of the network theory of implicit attitude. Finally, The IAT used in our study shows satisfactory psychometric properties in terms of internal consistency.

One of the limitations in the present study is that all participants are males, which requires more generalization if being applied to the general population. We recruited male smokers and non-smokers to minimize potential confounding factors. It has been demonstrated that gender effects exist in reactions to smoking-related cues [47–49]. However, no study has shown gender differences in implicit beliefs and behavioral tendencies. Thus, future research can dig into this interesting field. Our second limitation is that all participants are undergraduate students majoring in clinical medicine, who are at the age of desiring to become mature and independent, to be recognized, to interact with others. Their major can also be a confounding variable. These reasons make it difficult to expand our results to more general smoking groups. The third limitation is that we only recruit from one location. Further, the current study resides in the cross-sectional nature. The relationships found in this study are only correlational and our method does not permit inferences about causes and effects. Finally, the sample size in the present study is relatively small; hence, our data should be viewed as exploratory, and not conclusive prior to replication.

## Conclusions

In summary, this study is the first paper using one IAT to capture two dimensions of implicit attitude in groups of smokers and non-smokers. The results show that there are significant differences between smokers and non-smokers in implicit beliefs and behavioral tendencies toward smoking. In addition, there is a positive correlation between implicit beliefs and behavioral tendencies within smokers. This may provide a new perspective for measuring different dimensions of implicit attitudes by putting together these different dimensions of vocabulary into one implicit association test. Further, it may give some explanations for the mixed results in measuring automatic processes among smokers. The present study might be beneficial to the development of the network theory of implicit attitude. Moreover, it shows for the first time that implicit behavioral tendencies measured by IAT are related to smoking behaviors. This may provide some guidelines for smoking cessation interventions from an implicit level.

## Additional file

Additional file 1: Details of survey questionnaire, including demographics, smoking characteristics and the Fagerström test for nicotine dependence of participants. (DOCX 13 kb)

## Data Availability

The datasets used and/or analyzed during the current study are available from the corresponding author on reasonable request.

## Abbreviations

FTND: Fagerström test for nicotine dependence
IAT: Implicit association test
IB: Implicit beliefs
IBT: Implicit behavioral tendencies
