# Supplementary material for: The implicit beliefs and implicit behavioral tendencies towards smoking-related cues among Chinese male smokers and non-smokers
Source: BMC Public Health. 2019 Jul 25;19:1000. doi: 10.1186/s12889-019-7319-7 (PMC6659297; doi:10.1186/s12889-019-7319-7)
Supplement: Supplementary file 1 — Details of survey questionnaire, including demographics, smoking characteristics and the Fagerström test for nicotine dependence of participants. (DOCX 13 kb) [file 12889_2019_7319_MOESM1_ESM.docx]

**Details of survey questionnaire, including demographics, smoking characteristics and the Fagerström test for nicotine dependence of participants.**

| **Name** |  |
| --- | --- |
| **Age** |  |
| **Gender** |  |
| **Level of education (years)** |  |
| **Years of smoking** |  |
| **Cigarettes / day** |  |

**The Fagerström test for nicotine dependence (FTND) can be found from the reference as follow.**

Heatherton TF, Kozlowski LT, Frecker RC, Fagerstrom KO. The Fagerström test for nicotine dependence: A revision of the Fagerström tolerance questionnaire. Brit J Addict. 1991;86(9):1119–27.
